# Viral hepatitis knowledge and vaccination awareness among men who have sex with men (MSM) in 43 countries of the WHO European Region: results from the European MSM Internet Survey, EMIS-2017

**DOI:** 10.2807/1560-7917.ES.2024.29.45.2400099

**Published:** 2024-11-07

**Authors:** Sofia Burdi, Michael Brandl, Ulrich Marcus, Erika Duffell, Ettore Severi, Antons Mozalevskis, Kristi Rüütel, Achim Dörre, Axel J Schmidt, Sandra Dudareva

**Affiliations:** 1 Department of Infectious Disease Epidemiology, Robert Koch Institute (RKI), Berlin, Germany; 2 Postgraduate Training for Applied Epidemiology, Department of Infectious Disease Epidemiology, Robert Koch Institute (RKI), Berlin, Germany; 3 ECDC Fellowship Programme, Field Epidemiology path (EPIET), European Centre for Disease Prevention and Control (ECDC), Stockholm, Sweden; 4 Charité – Universitätsmedizin Berlin, corporate member of Freie Universität Berlin and Humboldt-Universität zu Berlin, Berlin, Germany; 5 European Centre for Disease Prevention and Control (ECDC), Stockholm, Sweden; 6 World Health Organization (WHO) Regional Office for Europe, Copenhagen, Denmark; 7 Department of Risk Behaviour Studies, National Institute for Health Development, Tallinn, Estonia; 8 Sigma Research, Department of Public Health, Environments and Society, London School of Hygiene and Tropical Medicine (LSHTM), London, United Kingdom

**Keywords:** hepatitis A, hepatitis B, men who have sex with men, surveys and questionnaires, Europe

## Abstract

**Background:**

Recent hepatitis A virus outbreaks in Europe affecting men who have sex with men (MSM) and ongoing hepatitis B virus transmission among MSM underscore the ongoing need for viral hepatitis prevention in this population.

**Aim:**

To describe viral hepatitis knowledge and associated factors among MSM in the WHO European Region to inform targeted prevention.

**Methods:**

In the European MSM Internet Survey (EMIS-2017), basic knowledge was defined as correctly identifying at least 4 of 5 statements about viral hepatitis and vaccination. We described basic knowledge by country. In a multilevel logistic regression model, we estimated adjusted odds ratios (aOR) with 95% confidence intervals (CI) for having basic knowledge and explanatory variables: sociodemographic characteristics, history of hepatitis C and/or HIV diagnosis, sexual orientation disclosure at last sexually transmitted infections (STI) test and outness.

**Results:**

Of 113,884 participants across 43 WHO European Region countries, 68% demonstrated basic knowledge, ranging from 50% in Israel to 80% in the Netherlands. Basic knowledge was significantly associated with older age (≥ 40 years vs < 25 years, aOR: 2.9, 95% CI: 2.7–3.0), a history of hepatitis C and/or HIV diagnosis (aOR: 1.8, 95% CI: 1.7–1.9) and sexual orientation disclosure at last STI test (aOR: 1.3, 95% CI: 1.2–1.3), among other factors.

**Conclusions:**

We found a knowledge disparity regarding viral hepatitis and hepatitis vaccination awareness among MSM across Europe, highlighting a need to address these gaps. A non-judgemental, accepting climate that allows individuals attending medical services to safely disclose their sexual orientation is fundamental to enable healthcare professionals to target information and preventative measures more effectively.

**Key public health message**

**What did you want to address in this study and why?**

Hepatitis A and B viruses cause liver inflammation, and infections can be prevented through vaccination. Greater knowledge of these diseases may lead to higher vaccination uptake. Men who have sex with men (MSM) are more likely to get infected with hepatitis A and B. We studied what MSM in the WHO European Region know about viral hepatitis and vaccination, and factors associated with better knowledge to improve prevention efforts.

**What have we learnt from this study?**

Two thirds of MSM in Europe had a basic understanding of viral hepatitis, but knowledge levels varied across countries. Respondents were generally more knowledgeable if they were older, had other health conditions such as hepatitis C or HIV infections, and were willing to disclose their sexual orientation to medical staff. Around 60% of unvaccinated MSM reported that they had never actively been offered either hepatitis A or B vaccination.

**What are the implications of your findings for public health?**

To bridge existing knowledge gaps, it is essential to provide information to people in risk populations like MSM. A simple but important step is to offer hepatitis A and B vaccines to all MSM seeking healthcare, especially when they are tested for HIV. Additionally, creating a supportive healthcare environment where individuals feel safe sharing their sexual orientation without fear of judgment is essential for this approach to be effective.

## Introduction

The World Health Organization (WHO) set the goal to eliminate viral hepatitis by 2030 [1] and defined men who have sex with men (MSM) as one of the key populations for viral hepatitis elimination [2]. MSM are at greater risk of viral hepatitis infection, exacerbated by stigma and discrimination from both the general population and healthcare professionals, resulting in lower access to essential viral hepatitis services [3,4]. The dynamics of viral hepatitis epidemics are shaped by vulnerabilities among key populations such as MSM [2] and thus prevention, diagnosis and treatment of viral hepatitis in this key population is central to its elimination.

Vaccines against hepatitis A and B have been widely available globally since the 1990s [5,6]. The WHO had advised countries to include universal hepatitis B vaccination into national immunisation programs by 1997 [5], and consequently, it has been increasingly adopted [7]. In contrast, many countries have no universal vaccination recommendation for hepatitis A [6,8]. Sex between men is still the second-most commonly reported route of transmission for acute hepatitis B case notifications in Europe [9]. Nevertheless, only about three quarters of European countries explicitly recommend hepatitis B vaccination for MSM [10]. Outbreaks of hepatitis A have been widely reported among MSM worldwide, such as a large multi-country hepatitis A outbreak impacting many European countries in 2016–18 [11,12]. But only just under half of European countries have established hepatitis A vaccination recommendations for MSM [10]. This demonstrates the ongoing need for prevention efforts in this population, including increasing the vaccination rates for both hepatitis A and B in MSM.

Enhancing knowledge and ensuring the availability of accurate information are crucial steps towards behaviour change, such as increasing vaccine acceptance [13,14]. While the current levels of knowledge about viral hepatitis among MSM may not be fully understood, making information readily accessible can help individuals make informed decisions. A study of HIV-negative MSM showed that common reasons for not being vaccinated against hepatitis B were their lack of knowledge about the existence of a vaccine to prevent hepatitis B infection and the misconception that they were not eligible for vaccination [15]. Knowledge about viral hepatitis in the WHO European Region, specifically among MSM, is poorly studied and the existing literature primarily focuses on the general population or other target groups [16-19]. We aimed to describe knowledge about viral hepatitis and vaccination awareness among MSM across Europe using data collected as part of the European MSM Internet Survey (EMIS) 2017, and to analyse factors associated with knowledge to inform targeted prevention practices.

## Methods

### Data sources

EMIS-2017 was an anonymous, cross-sectional online survey to assess the sexual health of MSM in Europe and to generate data for planning and monitoring prevention programmes. The survey was available in 33 languages and 50 predominantly European countries from 18 October 2017 to 31 January 2018, and included questions on demographics, sexual orientation and its disclosure, history of HIV and viral hepatitis, sexual behaviour, knowledge of hepatitis and vaccination, and access to healthcare services (countries listed in [20]). The survey was promoted through MSM dating apps, websites and across social media. 

Inclusion criteria for this study were being of or above the legal age for homosexual consent in the country of residence, informed consent to participate in the survey, residing in one of the 50 countries, identifying as man including trans man, and being sexually attracted to men and/or engaged in sexual activity with men. A total of 127,792 men met these criteria. Responses from microstates were included in the neighbouring national datasets: Andorra with Spain, Liechtenstein with Switzerland, Monaco with France, and San Marino with Italy. Respondents from Canada, the Philippines and Lebanon were excluded, resulting in data from 43 countries. From the total of 127,535 respondents residing in 43 countries of the WHO European Region, we further excluded 13,651 men who answered in more than one place in logically inconsistent ways, arriving at a final dataset of 113,884 responses. The European MSM Internet Survey (EMIS-2017) was developed through a comprehensive consultation process and methods have been described in detail elsewhere [20,21].

### Variables of interest

We created our main outcome variable ‘basic knowledge’ about viral hepatitis based on several true specific statements about viral hepatitis and hepatitis vaccination included in the EMIS-2017 questionnaire: (1) There are several types of hepatitis viruses, named after the letters of the alphabet; (2) Vaccines exist for both hepatitis A and hepatitis B; (3) ‘Hepatitis’ is an inflammation of the liver; (4) Most hepatitis is caused by viruses; (5) Doctors recommend MSM are vaccinated against both hepatitis A and hepatitis B viruses.

For each specific knowledge statement, participants were asked to choose one of the following response options: (1) I knew this already; (2) I wasn’t sure about this; (3) I didn’t know this already; (4) I don’t understand this; (5) I do not believe this.

An additive knowledge score was created based on the number of specific knowledge statements participants identified as known, i.e. answering (1) I knew this already. This score ranged from 0 to 5. Knowledge scores for participants who left at least one of the five specific knowledge statements unanswered were coded as missing. In order to dichotomise participants’ knowledge levels, we used the median as a reference point and defined a basic knowledge about viral hepatitis as having a knowledge score of at least 4 of 5, excluding missing values. By setting this threshold, we aimed to focus on participants with a foundational understanding of the topic.

We assessed the vulnerability of infection based on the question of whether the respondents were vaccinated against hepatitis A and B, respectively. Participants could choose one of the following responses: (1) No, because I've had hepatitis A/B (and am now naturally immune); (2) No, and I don't know if I'm immune; (3) No, I have chronic hepatitis B infection (hepatitis B only); (4) Yes, and I completed the course; (5) Yes, but I did not complete the course; (6) Yes, but I did not respond to the vaccinations; (7) I don't know.

We defined participants as vulnerable to hepatitis A/B if they reported ‘No, and I don't know if I'm immune’, ‘Yes, but I did not complete the course’ or ‘I don't know’. Vulnerable participants were subsequently asked whether they knew where to get vaccinated against hepatitis A/B. Another vaccination access awareness question was presented to all participants: whether they have been offered any hepatitis vaccination by a health service.

Other variables of interest, used in the descriptive analysis and as independent explanatory variables in regression models, were selected before the analyses began based on theoretical assumptions and published evidence [19,22-26]. These variables include sociodemographic variables, diagnosis of hepatitis C and/or HIV (as a proxy for frequent healthcare system contact), and sexual orientation disclosure variables, as a reflection of the social climate surrounding the survey participants. Additionally, we included a binary variable ‘Living in a country with hepatitis A and B vaccination recommendation for MSM (yes/no)’ to indicate whether participants reside in one of the 18 countries with hepatitis A and B vaccination recommendations specifically for MSM (Austria, Belgium, Bulgaria, Estonia, France, Germany, Greece, Iceland, Ireland, Israel, Italy, Luxembourg, the Netherlands, Norway, Portugal, Spain, Switzerland, the United Kingdom (UK)) [10].

Sociodemographic variables included age, which was collected as a continuous variable and then categorised into age groups (< 25 years, 25–39 years, ≥ 40 years), educational level, based on the number of years in full-time education beyond the age of 16 years (low: 0–1 year post age 16 years; mid: at least upper secondary, 2–5 years post age 16 years; high: first stage of tertiary or more, ≥ 6 years post age 16 years), financial coping (struggling/really struggling on present income, neither comfortable nor struggling on present income, living comfortably/really comfortably on present income), and settlement size (medium-sized or smaller settlements: < 500,000 inhabitants, big to very big cities: 500,000 or more inhabitants). Sexual orientation disclosure variables were sexual orientation disclosure at last sexual transmitted infections (STI) test (no/unsure, not asked because respondent did not undergo STI testing within the previous 12 months, yes definitely/probably) and outness, referring to the disclosure of one’s sexual orientation to family, friends and colleagues (out to no one or few, out to some, out to all or almost all) as a proxy for structural anti-gay stigma at population level [27].

### Statistical analysis

For each analysis, participants with missing values in the relevant variables were excluded. We described participants’ knowledge of the five specific viral hepatitis statements, basic knowledge and hepatitis vaccination access awareness by vulnerability to hepatitis A and B, respectively. We created a map to display percentages of basic knowledge by country of residency. Additionally, we described participants by age, history of hepatitis C and/or HIV diagnosis, education, financial situation, settlement size, sexual orientation disclosure at last STI test, outness, vulnerability to hepatitis A and B, and living in a country with hepatitis A and B vaccination recommendation for MSM stratified by basic knowledge.

For continuous variables, median and interquartile range (IQR), and for nominal variables, counts and percentages were calculated, excluding missing data. Using the Welch Two Sample t-test for continuous variables and the Pearson’s chi-square test for categorical variables we compared non-vulnerable and vulnerable participants, as well as participants with and without basic viral hepatitis knowledge. A p value < 0.05 was considered statistically significant.

A cascade of hepatitis vaccination knowledge was built showing proportions of vulnerable participants with knowledge about MSM-specific vaccination recommendations and, among them, the proportion with knowledge about where to receive vaccination for hepatitis A and B, respectively.

To investigate the association of explanatory variables with viral hepatitis knowledge, we applied univariable and multivariable multilevel logistic regression models. The knowledge score was dichotomised into the binary outcome variable basic viral hepatitis knowledge (yes/no), because the original variable did not meet the assumptions necessary for linear regression analysis. We selected explanatory variables guided by theoretical assumptions and supported by published evidence [15,18-22]. This ensures our analysis captures relevant variables while avoiding potential collinearity, thereby maintaining robustness and interpretability in our findings. Multilevel logistic regression analysis with a random intercept at the country level was performed to account for the hierarchical structure of the data and potential latent heterogeneity between countries. Univariable logistic regression models were considered to identify variables with statistically significant association with the outcome. Explanatory variables that yielded the largest model fit improvement, as determined by the likelihood ratio test, were added stepwise to the model.

To explore potential effect modification by country, a stratified analysis was performed. This involved fitting separate multivariable logistic regression models for each country while maintaining the same individual-level explanatory variables across strata. The effect estimates largely overlapped across countries and were included in the final model as fixed effects. Predictive accuracy of the final model was assessed by comparing predicted values to the actual values for each country.

The adjusted odds ratios (aORs) and the corresponding 95% confidence interval (95% CI) for factors associated with basic knowledge were estimated based on the final model and the intraclass correlation coefficient (ICC) was calculated.

Knowledge scores for participants with unanswered knowledge statements were coded as missing and were not included in the outcome basic knowledge. We compared characteristics of included (no missing data in knowledge statements) with excluded (missing data in knowledge statements) participants. We performed four sensitivity analyses regarding the multivariable multilevel regression models. In sensitivity analysis 1, the outcome was a knowledge score of 4–5, similar to the original model. However, the score was calculated based on all responses to the knowledge statements, including missing values that were coded as ‘not known’. To evaluate the robustness of the findings with respect to the chosen cut-off point of the knowledge score used to dichotomise the outcome variable basic knowledge, sensitivity analysis 2 was performed. This involved applying an alternative cut-off of 3 or higher compared with 4 or higher, as used in the original model. Responses to statement 5 “Doctors recommend MSM are vaccinated against both hepatitis A and hepatitis B” may vary based on the country-specific recommendations. To evaluate, whether our results remain robust with respect to this statement, we conducted sensitivity analysis 3, which involved excluding this statement from the outcome variable. In sensitivity analysis 4, we used the knowledge of statement 5 as the outcome.

Analyses were performed using R (version 4.1.3). Maps were created using the European Centre for Disease Prevention and Control (ECDC) Map Maker tool (EMMa; https://geoportal.ecdc.europa.eu/mapmaker).

## Results

### Knowledge about viral hepatitis and hepatitis vaccination awareness

**Figure 1:**
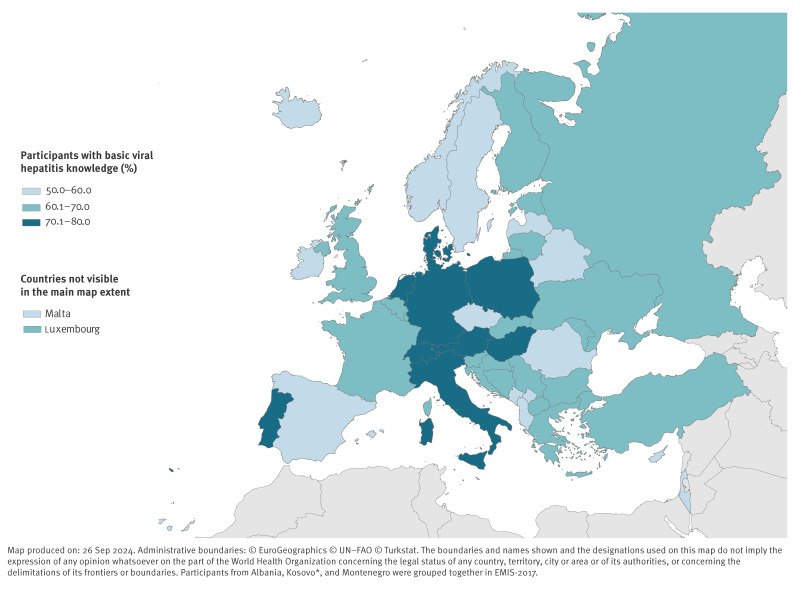
Percentage of participants with basic knowledge about viral hepatitis by country of residency in 43 countries of the WHO European Region, EMIS-2017 (n = 112,172)

EMIS-2017: European MSM Internet Survey; WHO: World Health Organization.

The median number of hepatitis statements known was 4 of 5. Of the respondents, 45% (50,815/112,172) knew all of the five facts and 3% (2,864/112,172) did not know any of the five facts. The fact most commonly known was that there are several types of hepatitis viruses, named after the letters of the alphabet (92%, 104,319/113,144) (Table 1). The least commonly known fact was that doctors recommend that MSM should be vaccinated against both hepatitis A and B (59%, 66,627/113,433). Most participants (83%, 94,357/113,272) knew that vaccines exist for both hepatitis A and B. Overall, 68% (76,242/112,172) of participants had basic knowledge about viral hepatitis. The proportion of participants with basic knowledge was highest in the Netherlands (80%, 2,758/3,446) and lowest in Israel (50%, 545/1,081) (Figure 1).

**Table 1:** Percentage of participants with specific knowledge, basic viral hepatitis knowledge and vaccination access awareness by vulnerability to hepatitis A/hepatitis B in 43 WHO European Region countries, EMIS-2017 (n = 113,884)

Item	MSM vulnerable^a^ to hepatitis A		MSM vulnerable^a^ to hepatitis B		MSM not vulnerable^a^ to hepatitis A		MSM not vulnerable^a^ to hepatitis B		Overall	
	%	n/N	%	n/N	%	n/N	%	n/N	%	n/N
**Specific knowledge (five knowledge statements)**										
There are several types of hepatitis viruses, named after the letters of the alphabet^b^	89	52,726/59,259	88	47,267/53,731	96	51,283/53,536	96	56,794/59,112	92	104,319/113,144
Vaccines exist for both hepatitis A and hepatitis B^b^	73	43,508/59,297	71	38,372/53,771	94	50,561/53,622	94	55,747/59,196	83	94,357/113,272
‘Hepatitis’ is an inflammation of the liver^b^	70	41,527/59,422	68	36,673/53,902	83	44,791/ 53,675	84	49,698/59,242	76	86,616/ 113,464
Most hepatitis is caused by viruses^b^	69	41,082/59,265	68	36,365/53,763	83	44,304/53,564	83	49,067/59,115	76	85,663/113,184
Doctors recommend MSM are vaccinated against both hepatitis A and B^b^	41	24,612/59,397	38	20,470/53,877	78	41,812/53,681	78	45,992/59,253	59	66,627/113,433
**Basic knowledge**										
Basic knowledge^b^ (knowledge score: 4–5)	55	32,276/58,761	52	27,631/53,264	82	43,728/53,080	83	48,415/58,620	68	76,242/112,172
**Vaccination access awareness**										
Know where to vaccinate against hepatitis A/hepatitis B	46	27,352/59,179	45	24,234/53,861	Not asked		Not asked		NA	
Know about MSM specific vaccination recommendations and where to vaccinate	26	15,467/59,068	24	12,783/53,762	Not asked		Not asked		NA	

EMIS-2017: European MSM Internet Survey; MSM: men who have sex with men; NA: not applicable; WHO: World Health Organization.

^a^ Being vulnerable is defined as not being (fully) vaccinated, not immune or not aware of vaccination status.

^b^ P value < 0.001 comparing MSM vulnerable to hepatitis A with MSM not vulnerable to hepatitis A, and MSM vulnerable to hepatitis B with MSM not vulnerable to hepatitis B, respectively for five knowledge statements and basic knowledge, based on Pearson’s chi-square test.

* This designation is without prejudice to positions on status and is in line with United Nations Security Council Resolution 1244/99 and the International Court of Justice Opinion on the Kosovo Declaration of Independence.

Overall, 53% (59,510/113,303) of EMIS-2017 participants were vulnerable to hepatitis A and 48% (53,982/113,356) to hepatitis B. The percentage of participants with specific and basic viral hepatitis knowledge was higher in non-vulnerable MSM (vaccinated/immune) participants compared with vulnerable participants (Table 1).

Of respondents vulnerable to hepatitis A and B, 58% (34,570/59,342) and 62% (33,404/53,841), respectively, had never been offered any hepatitis vaccination.

Participants with higher knowledge scores showed higher proportions of older age groups, individuals with a hepatitis C and/or HIV diagnosis, individuals with a higher level of education, those with a comfortable financial situation, residents of larger cities, individuals who had disclosed their sexual orientation at the last STI test, those who had come out to almost everyone, those not vulnerable to hepatitis A and hepatitis B, respectively and those living in countries with hepatitis A and B vaccination recommendations for MSM compared with those with lower knowledge scores (Table 2). The median age of all included participants was 36 years (IQR: 27–47). The median age of participants with a knowledge score of 0–3 was 30 years (IQR: 23–41), whereas the median age of participants with a knowledge score of 4–5 was 38 years (IQR: 29–48). The mean age difference between the two knowledge groups was statistically significant (p < 0.001).

**Table 2:** Characteristics of participants by knowledge score in 43 WHO European Region countries, EMIS-2017 (n = 112,172)

Characteristics	Basic knowledge = No Knowledge score: 0–3 n = 35,930		Basic knowledge = Yes Knowledge score: 4–5 n = 76,242		Total n = 112,172	
	n	%	n	%	n	%
**Age group in years** ^a^						
< 25	10,534	29	10,393	14	20,927	19
25–39	16,097	45	32,823	43	48,920	44
≥ 40	9,299	26	33,026	43	42,325	38
**Ever diagnosed with hepatitis C or HIV** ^a^						
No	31,754	94	63,911	86	95,665	89
Yes	1,906	5.7	10,393	14	12,299	11
Missing	2,270		1,938		4,208	
**Education** ^a^						
Low (0–1 year post age 16 years)	2,174	6.5	2,955	4.2	5,129	4.9
Mid (at least upper secondary; 2–5 years post age 16 years)	14,286	43	23,703	33	37,989	36
High (first stage of tertiary or more; ≥ 6 years post age 16 years)	16,883	51	44,476	63	61,359	59
Missing	2,587		5,108		7,695	
**Financial coping** ^a^						
Struggling/really struggling on present income	7,117	20	11,722	15	18,839	17
Neither comfortable nor struggling on present income	13,512	38	24,375	32	37,887	34
Living comfortably/really comfortably on present income	15,083	42	39,898	53	54,981	49
Missing	218		247		465	
**Settlement size** ^a^						
Medium-sized or smaller settlements (< 500,000)	26,164	74	51,867	69	78,031	70
Big to very big cities (≥ 500,000)	9,371	26	23,556	31	32,927	30
Missing	395		819		1,214	
**Sexual orientation disclosure at last STI test** ^a^						
No/unsure	3,555	9.9	8,243	11	11,798	11
No STI test in the previous 12 months (not asked)	24,430	68	38,816	51	63,246	56
Yes	7,936	22	29,154	38	37,090	33
Missing	9		29		38	
**Outness** ^a^						
Out to none or few	12,474	35	20,318	27	32,792	29
Out to some	10,599	30	21,094	28	31,693	28
Out to (almost) all	12,292	34	34,058	45	46,350	41
Missing	565		772		1,337	
**Vulnerable to hepatitis A** ^a, b^						
No	9,352	26	43,728	58	53,080	47
Yes	26,485	74	32,276	42	58,761	53
Missing	93		238		331	
**Vulnerable to hepatitis B** ^a, b^						
No	10,205	28	48,415	64	58,620	52
Yes	25,633	72	27,631	36	53,264	48
Missing	92		196		288	
**Living in a country with hepatitis A and B vaccination recommendation for MSM** ^a^						
No	10,320	29	18,519	24	28,839	26
Yes	25,610	71	57,723	76	83,333	74

EMIS-2017: European MSM Internet Survey; HIV: human immunodeficiency virus; MSM: men who have sex with men; STI: sexually transmitted infection.

^a^ All p values < 0.001; comparison of participants with basic knowledge = no versus basic knowledge = yes, based on Pearson's chi-square test.

^b^ Being vulnerable is defined as not being (fully) vaccinated, not immune or not aware of vaccination status.

### Cascade of hepatitis vaccination knowledge

**Figure 2:**
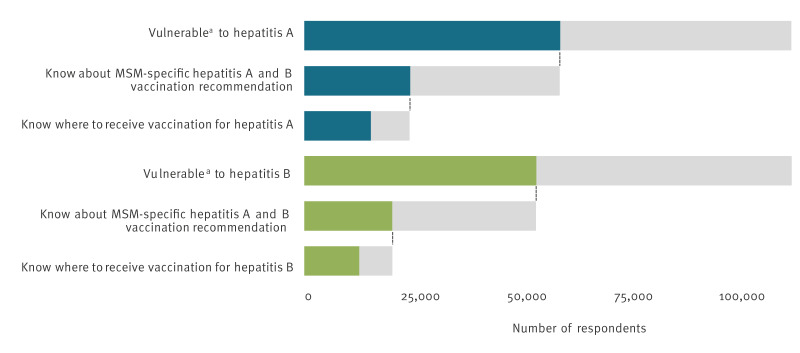
Cascade of knowledge about hepatitis vaccination among vulnerable participants in 43 WHO European Region countries, EMIS-2017 (n =113,303 for hepatitis A and n = 113,356 for hepatitis B)

EMIS-2017: European MSM Internet Survey; MSM: men who have sex with men; WHO: World Health Organization.

^a^ Being vulnerable is defined as not being (fully) vaccinated, not immune or not aware of vaccination status.

Figure 2 illustrates the sequential awareness levels of hepatitis A and B vaccination information in vulnerable participants, showing that less than half of vulnerable participants (hepatitis A: 24,612/59,397; hepatitis B: 20,470/53,877) knew about the MSM-specific recommendations and 63% (hepatitis A: 15,467/24,457; hepatitis B: 12,783/20,425) of those knew where to receive the vaccination.

Blue and green bars represent data for hepatitis A and B, respectively. Shaded bars indicate the number of vulnerable/knowledgeable participants. Grey bars indicate number of non-vulnerable/unaware participants. Missing data have been excluded.

### Factors associated with basic viral hepatitis knowledge

The odds for basic viral hepatitis knowledge increased with age, a history of diagnosed hepatitis C and/or HIV, educational attainment, income comfortability, settlement size, sexual orientation disclosure at the last test for STI and outness (Table 3). Chances of having basic viral hepatitis knowledge were 2.9 times higher for participants aged ≥ 40 years compared with participants aged < 25 years. MSM with a history of HIV and/or hepatitis C had almost twice the odds (aOR: 1.8) of having basic viral hepatitis knowledge compared with those without. Furthermore, participants who did not undergo STI testing in the previous 12 months had 40% lower odds of having basic viral hepatitis knowledge compared with those who did undergo STI testing, whereas those who disclosed their sexual behaviour during their last STI test had 1.3 times higher odds. Approximately 3% of the total variance in hepatitis knowledge can be attributed to between-country variability (ICC: 0.03). The final model exhibited a predictive accuracy of 71% with respect to the binary outcome basic knowledge and a predictive accuracy of 60–81% when testing for each country separately.

**Table 3:** Univariable and multivariable multilevel analysis for basic viral hepatitis knowledge in 43 WHO European Region countries, EMIS-2017 (n = 98,347)

Characteristics	Univariable		Multivariable		p value
	OR	95% CI	aOR	95% CI	
**Age group in years**					
< 25	Ref.				
25–39	2.00	1.93–2.07	1.52	1.46–1.58	< 0.001
≥ 40	3.55	3.42–3.69	2.85	2.72–2.96	< 0.001
**Ever diagnosed with hepatitis C and/or HIV **					
No	Ref.				
Yes	2.72	2.58–2.87	1.76	1.66–1.86	< 0.001
**Education **					
Low (0–1 year post age 16 years)	Ref.				
Mid (at least upper secondary; 2–5 years post age 16 years)	1.21	1.13–1.28	1.30	1.21–1.38	< 0.001
High (first stage of tertiary or more; ≥ 6 years post age 16 years)	1.91	1.79–2.03	1.72	1.61–1.84	< 0.001
**Financial coping**					
Struggling/really struggling on present income	Ref.				
Neither comfortable nor struggling on present income	1.09	1.05–1.14	1.09	1.05–1.14	< 0.001
Living comfortably/really comfortably on present income	1.59	1.54–1.66	1.37	1.32–1.43	< 0.001
**Settlement size **					
Medium-sized or smaller settlements (< 500,000)	Ref.				
Big to very big cities (≥ 500,000)	1.26	1.23–1.30	1.10	1.06–1.14	< 0.001
**Sexual disclosure at last STI test**					
No/unsure	Ref.				
No STI test in the previous 12 months (not asked)	0.69	0.66–0.73	0.60	0.57–0.63	< 0.001
Yes	1.58	1.50–1.66	1.25	1.19–1.32	< 0.001
**Outness**					
Out to none or few	Ref.				
Out to some	1.21	1.16–1.25	1.13	1.09–1.17	< 0.001
Out to (almost) all	1.66	1.61–1.72	1.42	1.37–1.47	< 0.001
**Living in a country with hepatitis A and B vaccination recommendation for MSM **					
No	Ref.				
Yes	1.24	1.20–1.27	0.83	0.68–1.02	0.073

CI: confidence interval; HIV: human immunodeficiency virus; MSM: men who have sex with men; OR: odds ratio; Ref.: reference; STI: sexually transmitted infection.

Basic viral hepatitis knowledge was defined as a knowledge score: 4–5.

A total of 1,712 participants’ knowledge scores were coded as missing, as those participants left at least one of the five statements unanswered and were thus excluded from the outcome. Supplementary Table S1 shows that a higher proportion of excluded participants had no basic viral hepatitis knowledge, were > 40 years of age, not out or only out to few, had not taken an STI test in the previous 12 months and were living in a country with no hepatitis A and B vaccination recommendation for MSM. However, sensitivity analysis 1 showed that our results were consistent when participants with any missing knowledge responses were included in the multivariable multilevel regression analysis, as illustrated in Supplementary Table S2. Additionally, sensitivity analysis 2 indicated that our findings remained robust when using a lower cut-off score for dichotomising the outcome variable. Furthermore, our analyses confirmed that including statement 5 (‘Doctors recommend are vaccinated against both hepatitis A and B’) was appropriate, as the results remained consistent whether this statement was included or excluded. Full results for sensitivity analyses can be found in Supplementary Table S2.

## Discussion

We studied knowledge about viral hepatitis and vaccination access in over 110,000 MSM across 43 WHO European Region countries through an online survey that was conducted during the aftermath of a large European hepatitis A outbreak that predominantly impacted MSM [11]. We found about two thirds of MSM had fundamental knowledge about viral hepatitis. We observed geographic differences in viral hepatitis knowledge among MSM across Europe and knowledge gaps regarding vaccination recommendations for MSM. We found that older men, those with higher educational level, a comfortable financial situation, living in big settlements, those who disclosed their sexual orientation in the healthcare context, were out to family and friends, and had regular contact with the health system because of diagnosed hepatitis C and/or HIV had higher chances of having basic knowledge about viral hepatitis. A substantial proportion of men who were aware of both the vaccination recommendations for MSM and where to obtain the vaccination had not received it, indicating possible missed opportunities for prevention, possibly because of a lack of proactive offers from healthcare providers. 

Knowledge about viral hepatitis showed regional and national differences. Reasons for the variation might include differences in education and awareness programs regarding viral hepatitis or differences in healthcare systems, i.e. availability of sexual health counselling and prevention services, official vaccination recommendations. Studies have shown that differences in health educational programs can contribute to disparities in disease awareness and understanding [28]. Different availability and accessibility of information such as sexual health resources may also play a role [29]. Additionally, stigma and experiences of discrimination, that can hinder access to information and healthcare services, could also contribute to the different knowledge levels between countries. A study on Black/African American MSM showed that perceived discrimination in healthcare settings can hinder awareness of HIV prevention [30]. Differences in public health measures to contain the hepatitis A outbreak predominantly affecting MSM may also be relevant in explaining knowledge disparities across European countries [11,12]. Given the knowledge disparities across Europe, it remains essential to provide comprehensive information about hepatitis, hepatitis prevention and vaccination recommendations throughout the region.

In this study, only just over half of participants were aware that vaccinations against hepatitis A and B are recommended for MSM. Based on viral hepatitis epidemiology in Europe [31], doctors should recommend vaccination to all MSM, irrespective of national guidelines. However, individual doctors’ recommendations may be influenced by country-specific vaccination guidelines. Among the 43 countries included in our study, Brandl et al. found that, at the time of EMIS-2017, hepatitis A vaccination was recommended for MSM in 19 countries, while hepatitis B vaccination in 32 countries [10]. Hepatitis A and B vaccination for MSM was recommended and free in seven countries (Germany, Greece, Ireland, Italy, Luxembourg, Spain and the UK) [10]. Knowledge of this recommendation is dependent on the chance of MSM to disclose their sexual orientation in healthcare settings. Nondisclosure may limit access to important health recommendations. Our data revealed a wide range in the extent to which participants disclosed their sexual orientation to healthcare providers during their last STI test from 24% in Russia to 96% in Malta (data not shown) [32].

Our findings indicate a potential link between higher knowledge and increased vaccine uptake: MSM who are protected from hepatitis A virus and hepatitis B virus infections through vaccination or previous infection had higher knowledge levels than those who are not. Vaccine acceptance, as described by the Strategic Advisory Group of Experts on Immunization (SAGE) Working Group on Vaccine Hesitancy, is influenced by multiple determinants, with knowledge being among the fundamental factors [13]. Studies analysing knowledge and hepatitis B vaccination status, as well as reasons for not receiving hepatitis B vaccination, have shown that hepatitis B vaccine acceptance is associated with knowledge about the disease and vaccination recommendations and lack of information was stated as one of several reasons for not being immunised [15,33-35]. Matthews et al. found that 28% of unvaccinated, HIV- and hepatitis B-negative MSM in a 2010 web-based survey in the United States stated that they didn’t know there was a vaccine that prevents hepatitis B virus infection [15].

A substantial number of unvaccinated MSM (hepatitis A: 26%; hepatitis B: 24%) in our study were aware of both the specific vaccination recommendations and where to receive vaccines for hepatitis A and hepatitis B but remained unvaccinated. This suggests that factors beyond knowledge influence vaccination uptake among MSM. Such factors may include perceived risk, costs, feasibility, and a lack of proactive vaccination efforts by healthcare providers, as described previously [15]. In fact, our data reveal that 58% of MSM vulnerable to hepatitis A and 62% of MSM vulnerable to hepatitis B have never been offered any hepatitis vaccination. This could be due to a number of structural- or provider-level factors such as healthcare providers not knowing or implementing vaccination recommendations for MSM, or MSM not disclosing their sexual orientation because of anticipated stigma [36,37]. Healthcare providers must be educated on vaccination recommendations for specific at-risk populations and understand which groups require targeted prevention efforts. Taking a patient’s sexual history during healthcare visits is vital for identifying vulnerable groups and delivering targeted prevention strategies to key populations. 

A relatively low intraclass correlation coefficient in our multivariable model indicates that individual-level differences play a larger role in explaining the variability in viral hepatitis knowledge than country-level variability. We observed that the odds for basic viral hepatitis knowledge was highest for older age, a diagnosis of hepatitis C and/or HIV, and higher educational attainment. Our findings differ from results of studies in the general population, where younger individuals tended to have higher knowledge levels, likely due to better access to information [17,19]. However, in our study among MSM, higher knowledge among older men might be explained by the fact that individuals often come out later in life. Consequently, the acquisition and exchange of information within the community also occur later and continue to develop over time. Furthermore, longer exposure to specific vaccination recommendations, including during healthcare visits, as well as a higher likelihood of present or past hepatitis A or hepatitis B virus infection can explain the association of older age with knowledge.

The association between being diagnosed with hepatitis C and/or HIV and having basic viral hepatitis knowledge may be attributed to several factors. Firstly, individuals with these diagnoses may be more motivated to self-educate on various health topics. Secondly, they have regular contact with health services, and hence greater access to health promotion. Regular contact with healthcare providers and prevention services should be made easily accessible, especially for key populations such as MSM. Such a regular contact is a valuable opportunity to inform, offer prevention services such as vaccination, test and treat diagnosed infections. It is also noteworthy that MSM who disclosed their sexual orientation at the last STI test had higher odds of basic knowledge compared with those who did not disclose. One possible explanation for this is that disclosing one’s sexual orientation allows healthcare providers to offer tailored health promotion and information, which can improve knowledge levels. Additionally, an environment with less stigma may facilitate disclosure, enhance communication and make it easier for patients to ask questions and receive accurate answers. Previous research has shown that MSM revealing their sexual orientation to healthcare providers is associated with receiving vaccination for hepatitis A and B and undergoing HIV/STI testing [38-41].

There are some limitations to our study. Firstly, although EMIS-2017 was able to reach a large number of MSM across many European countries, the sample may not be representative of the European MSM population. We lacked information regarding how the sample differs from the broader MSM population. The recruiting method may have led to the participation of MSM who are more sexually active and therefore have better knowledge. However, the process of collecting anonymous data online was efficient, cost-effective, and potentially reduced the influence of social desirability bias. Secondly, the analysed data are from 2017–18, predating the COVID-19 pandemic. COVID-19 has disrupted health services and prevention efforts [42], potentially affecting knowledge levels. As the EMIS-2017 was conducted during the aftermath of a hepatitis A outbreak primarily affecting MSM, results might have differed if the survey had been conducted later. Nevertheless, our study continues to hold relevance in evaluating viral hepatitis knowledge in the context of this outbreak in multiple European cities and in addressing the lack of comprehensive studies on this topic. Thirdly, all EMIS-2017 data were self-reported and therefore prone to recall bias. Also, hepatitis A and B viruses and vaccinations may have been confused by participants. Considering that hepatitis B is typically a childhood vaccination, while hepatitis A is not, we would have anticipated a more noticeable contrast between the proportions of MSM who are vulnerable to hepatitis A compared with those vulnerable to hepatitis B. Among the 43 countries included in our study, 40 have general vaccination recommendations for hepatitis B. The exceptions are Denmark, Finland, and Iceland. For hepatitis A, general vaccination recommendations are implemented in five countries: Cyprus, Greece, Israel, Russia and Türkiye. Additionally, Italy recommends vaccination for endemic areas, while Spain recommends it in one region (Catalonia) and two cities (Ceuta and Melilla) [7,8]. Thus, true vaccination rates for hepatitis B may be higher because younger participants might have been vaccinated as children and were not aware of it. Fourthly, while large surveys like EMIS also provide an opportunity for health promotion and participants — such as presenting participants with true statements about hepatitis and hepatitis vaccination and asking whether they knew them — the nature of the question may have prompted affirmative responses, even if participants did not actually know the statements. However, while this may have led to an over-estimation of knowledge levels overall and across countries, it is unlikely that it affected the associations presented in this study. Fifthly, the chosen outcome cut-off and exclusion of missing values in knowledge score calculation may have introduced bias. Nevertheless, sensitivity analyses confirmed the robustness of regression analysis results. Finally, the question of whether better knowledge also leads to preventive behaviour cannot be answered with this analysis. Questions on reasons for not vaccinating were not part of EMIS-2017.

## Conclusions

Addressing the knowledge disparities regarding viral hepatitis and hepatitis vaccination among MSM, along with the missed prevention opportunities identified in our study, is crucial for achieving the WHO elimination goals. To meet these goals, it is essential to strengthen the dissemination of comprehensive information on hepatitis and hepatitis prevention including vaccination, particularly targeting younger men in small settlements with a low level of education and poor financial resources. Additionally, offering hepatitis A and B vaccination to all MSM entering healthcare is an easy-to-accomplish yet much needed public health effort across Europe. Regular contact with healthcare providers and prevention services should be easily accessible, providing key opportunities to inform, vaccinate, test, and treat. Our data highlight the importance of healthcare providers taking a patient’s sexual history to identify vulnerable groups and to pass on targeted information and prevention recommendations to key populations. Certainly, this requires a non-stigmatising environment that allows men to disclose. Efforts to create a stigma-free environment for MSM across Europe must continue.

## *Note

This designation is without prejudice to positions on status and is in line with United Nations Security Council Resolution 1244/99 and the International Court of Justice Opinion on the Kosovo Declaration of Independence.

## Supplementary Data

237000 Supplement
