# Supplementary material for: Viral hepatitis knowledge and vaccination awareness among men who have sex with men (MSM) in 43 countries of the WHO European Region: results from the European MSM Internet Survey, EMIS-2017
Source: Euro Surveill. 2024 Nov 7;29(45):2400099. doi: 10.2807/1560-7917.ES.2024.29.45.2400099 (PMC11544719; doi:10.2807/1560-7917.ES.2024.29.45.2400099)
Supplement: Supplement [file 24-00099_BURDI_Supplement.pdf]

Supplement

This supplementary material is hosted by *Eurosurveillance* as supporting information alongside the article "self-reported hepatitis A and B vaccination coverage among men who have sex with men (MSM), associated factors, and vaccination recommendations in 43 countries of the WHO European Region: results from the European MSM Internet Survey, EMIS-2017", on behalf of the authors, who remain responsible for the accuracy and appropriateness of the content. The same standards for ethics, copyright, attributions and permissions as for the article apply. Supplements are not edited by *Eurosurveillance* and the journal is not responsible for the maintenance of any links or email addresses provided therein.

SUPPLEMENTARY FIGURE S1: Distribution of participants by country in 43 WHO European Region countries, EMIS-2017 (n = 113,884)

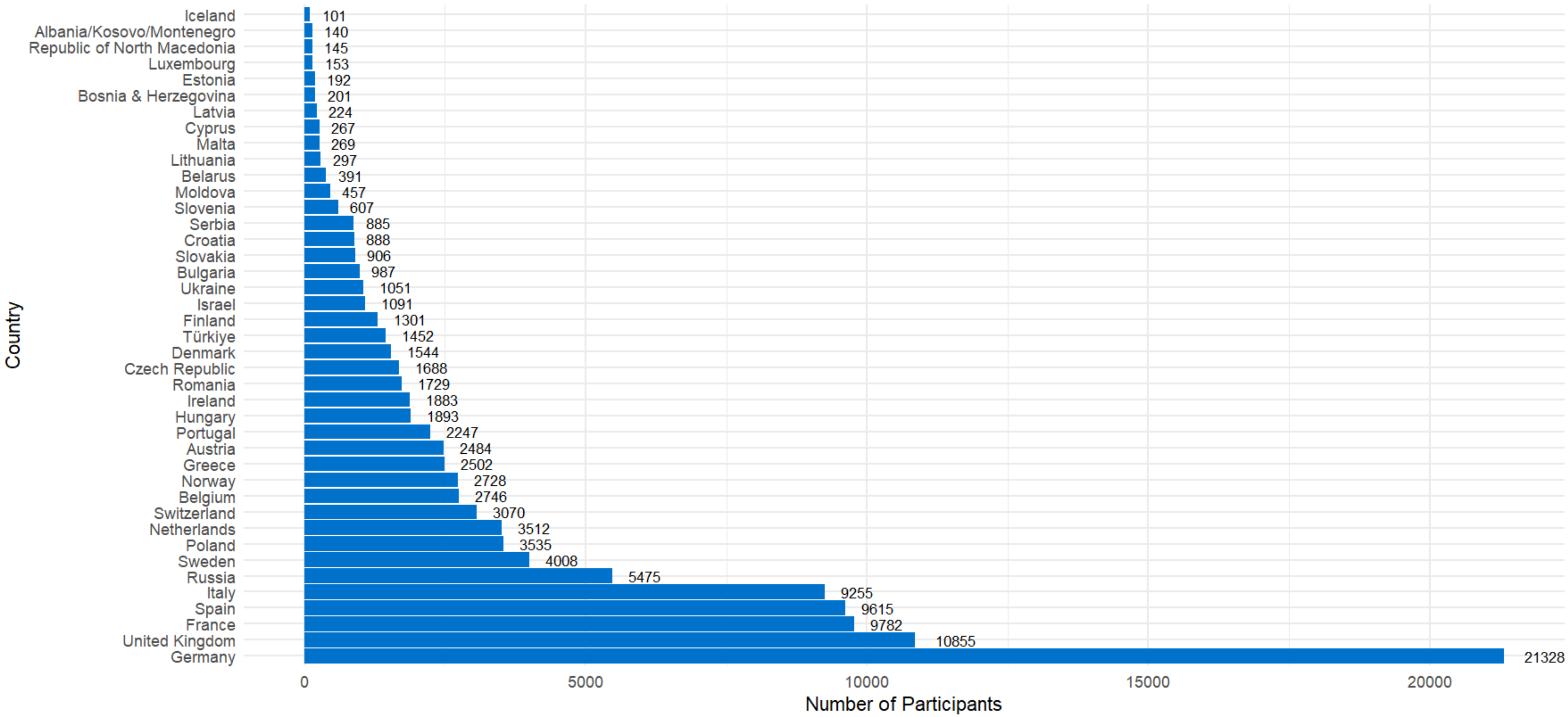

Albania, Kosovo* and Montenegro were grouped together in EMIS-2017.

* This designation is without prejudice to positions on status and is in line with United Nations Security Council Resolution 1244/99 and the International Court of Justice Opinion on the Kosovo Declaration of Independence.

SUPPLEMENTARY TABLE S1: Participants with and without missing data in five knowledge statements by different characteristics in 43 WHO European Region countries, EMIS-2017 (n = 113,884)

**Characteristics**	**Participants included in outcome (no knowledge statement missings) n (%)** N = 112,172	**Participants excluded in outcome (at least one knowledge statement missing) n (%)** N = 1,712	**p value[a]**	**Total n (%)** N = 113,884
**Basic knowledge (knowledge score: 4 – 5)**			<0.001	
Yes	76,242 (68%)	690 (40%)		76,932 (68%)
No	35,930 (32%)	1,022 (60%)		36,952 (32%)
**Age group in years**			<0.001	
<25	20,927 (19%)	243 (14%)		21,170 (19%)
25–39	45,934 (41%)	508 (30%)		46,442 (41%)
≥ 40	45,311 (40%)	961 (56%)		46,272 (41%)
**Ever diagnosed with hepatitis C or HIV**			0.3	
No	95,665 (89%)	1,266 (88%)		96,931 (89%)
Yes	12,299 (11%)	177 (12%)		12,476 (11%)
**Education**			0.074	

Characteristics	Participants included in outcome (no knowledge statement missings) n (%)<br>N = 112,172	Participants excluded in outcome (at least one knowledge statement missing) n (%)<br>N = 1,712	p value[a]	Total n (%)<br>N = 113,884
Low (0–1 year post age 16 years)	5,129 (4.9%)	84 (6.2%)		5,213 (4.9%)
Mid (at least upper secondary; 2–5 years post age 16 years)	37,989 (36%)	494 (37%)		38,483 (36%)
High (first stage of tertiary or more; ≥ 6 years post age 16 years)	61,359 (59%)	774 (57%)		62,133 (59%)
**Financial Coping**			0.4	
Struggling/really struggling on present income	18,839 (17%)	250 (16%)		19,089 (17%)
Neither comfortable nor struggling on present income	37,887 (34%)	521 (33%)		38,408 (34%)
Living comfortably/really comfortably on present income	54,981 (49%)	796 (51%)		55,777 (49%)
**Settlement Size**			0.15	
Medium-sized or smaller settlements (<500,000)	78,031 (70%)	1,193 (72%)		79,224 (70%)
Big to very big cities (≥ 500,000)	32,927 (30%)	464 (28%)		33,391 (30%)

Characteristics	Participants included in outcome (no knowledge statement missings) n (%) N = 112,172	Participants excluded in outcome (at least one knowledge statement missing) n (%) N = 1,712	p value[a]	Total n (%) N = 113,884
**Sexual orientation disclosure at last STI test**			<0.001	
No / unsure	11,798 (11%)	124 (7.3%)		11,922 (10%)
No STI test in the previous 12 months (not asked)	63,246 (56%)	1,147 (67%)		64,393 (57%)
Yes	37,090 (33%)	435 (25%)		37,525 (33%)
**Outness**			<0.001	
Out to none or few	32,792 (30%)	634 (38%)		33,426 (30%)
Out to some	31,693 (29%)	429 (26%)		32,122 (29%)
Out to (almost) all	46,350 (42%)	594 (36%)		46,944 (42%)
**Vulnerable[b] towards hepatitis A**			0.3	
No	53,080 (47%)	713 (49%)		53,793 (47%)
Yes	58,761 (53%)	749 (51%)		59,510 (53%)
**Vulnerable[b] towards hepatitis B**			0.4	
No	58,620 (52%)	754 (51%)		59,374 (52%)

Characteristics	Participants included in outcome (no knowledge statement missings) n (%) N = 112,172	Participants excluded in outcome (at least one knowledge statement missing) n (%) N = 1,712	p value[a]	Total n (%) N = 113,884
Yes	53,264 (48%)	718 (49%)		53,982 (48%)
**Living in a country with hepatitis A and B vaccination recommendation for MSM**			<0.001	
No	28,839 (26%)	514 (30%)		29,353 (26%)
Yes	83,333 (74%)	1,198 (70%)		84,531 (74%)

HIV: Human Immunodeficiency Virus; MSM: men who have sex with men; STI: sexually transmitted infection
[a] Pearson's Chi-square test
[b] Being vulnerable is defined as not being (fully) vaccinated, not immune or not aware of vaccination status.

SUPPLEMENTARY TABLE S2: Sensitivity analyses 1—4, multivariable multilevel regression models applied to different subsets of participants / with different outcomes, EMIS-2017

	Sensitivity Analysis 1[a]		Sensitivity Analysis 2[a]		Sensitivity Analysis 3[a]		Sensitivity Analysis 4[a]	
**Characteristics**	aOR	95% CI	aOR	95% CI	aOR	95% CI	aOR	95% CI
**Age group in years**								
<25	Ref.		Ref.		Ref.		Ref.	
25–39	1.51	1.46, 1.57	1.55	1.49, 1.63	1.52	1.46, 1.58	1.26	1.21, 1.31
≥ 40	2.79	2.67, 2.91	3.01	2.86, 3.17	2.85	2.73, 2.97	1.88	1.81, 1.96
**Ever diagnosed with hepatitis C and/or HIV**								
No	Ref.		Ref.		Ref.		Ref.	
Yes	1.76	1.67, 1.87	1.76	1.63, 1.91	1.76	1.66, 1.86	2.01	1.91, 2.12
**Education**								
Low (0–1 year post age 16 years)	Ref.		Ref.		Ref.		Ref.	
Mid (at least upper secondary; 2–5 years post age 16 years)	1.29	1.20, 1.37	1.39	1.29, 1.50	1.29	1.20, 1.38	1.13	1.06, 1.21
High (first stage of tertiary or more; ≥ 6 years post age 16 years)	1.71	1.60, 1.83	1.95	1.81, 2.11	1.72	1.61, 1.83	1.38	1.29, 1.48

**Financial coping**								
Struggling/really struggling on present income	Ref.		Ref.		Ref.		Ref.	
Neither comfortable nor struggling on present income	1.09	1.05, 1.14	1.13	1.08, 1.19	1.09	1.05, 1.14	1.10	1.05, 1.14
Living comfortably/really comfortably on present income	1.38	1.32, 1.43	1.43	1.36, 1.50	1.37	1.32, 1.43	1.46	1.40, 1.52
**Settlement size**								
Medium-sized or smaller settlements (<500,000)	Ref.		Ref.		Ref.		Ref.	
Big to very big cities (≥500,000)	1.10	1.06, 1.13	1.13	1.08, 1.18	1.10	1.06, 1.14	1.21	1.17, 1.25
**Sexual disclosure at last STI test**								
No / unsure	Ref.		Ref.		Ref.		Ref.	
No STI test in the previous 12 months (not asked)	0.60	0.57, 0.63	0.58	0.54, 0.61	0.60	0.57, 0.63	0.60	0.57, 0.62
Yes	1.25	1.18, 1.32	1.35	1.26, 1.45	1.25	1.19, 1.32	1.84	1.75, 1.93
**Outness**								
Out to none or few	Ref.		Ref.		Ref.		Ref.	
Out to some	1.14	1.10, 1.18	1.15	1.09, 1.20	1.13	1.09, 1.17	1.24	1.19, 1.28
Out to (almost) all	1.43	1.38, 1.48	1.48	1.41, 1.55	1.42	1.37, 1.47	1.68	1.62, 1.74

**Living in a country with hepatitis A and B vaccination recommendation for MSM**								
No	Ref.		Ref.		Ref.		Ref.	
Yes	0.83	0.68, 1.02	0.85	0.67, 1.07	0.83	0.68, 1.01	1.19	0.95, 1.50

aOR: Odds Ratio; CI: Confidence Interval; HIV: Human Immunodeficiency Virus; MSM: men who have sex with men; Ref.: Reference; STI: sexually transmitted infection

[a] Sensitivity Analysis 1 = multilevel multivariable analysis; dependent variable: knowledge of ≥4 out of 5 knowledge statements for all participants, including those with one or more missing values in knowledge statements

[a] Sensitivity Analysis 2 = multilevel multivariable analysis; dependent variable: knowledge of ≥ 3 out of 5 knowledge statements for all participants, excluding those with one or more missing values in knowledge statements

[a] Sensitivity Analysis 3 = multilevel multivariable analysis; dependent variable: knowledge of 4 out of 4 knowledge statements (without statement "Doctors recommend Men who have sex with men (MSM) are vaccinated against both hepatitis A and hepatitis B.") for all participants, excluding those with missing values in knowledge statement

[a] Sensitivity Analysis 4 = multilevel multivariable analysis; dependent variable: knowledge of statement "Doctors recommend MSM are vaccinated against both hepatitis A and hepatitis B" for all participants, excluding those with missing values in knowledge statements
